# Our Noble Profession

**Published:** 1891-06

**Authors:** 


### "OUR NOBLE PROFESSION."

This is the constant boast of medical men, and we have heard it from men of questionable standing, even. Scarcely can an address or set speech be made, but the hackneyed phrase is dragged in. That it is a noble profession as originally instituted,—made noble by those who live up to its principles, no one doubts; that some physicians are noble and self-sacrificing and unselfish,—that their real aim is to do good, cannot be denied; but that every man who boasts of "our noble profession" is "noble," or even a gentleman, cannot be admitted. In times of great distress, in epidemics, and in great calamities, such as at Johnstown, in wrecks, etc., the true nobility in the profession comes out, and the press is ready to sound its praises. But in all these years it seems that the profession has not been able to impress the people, at least the law makers, with any sense of their greatness, goodness, wisdom or "nobility." Witness the many failures to secure the pasyage of sanitary laws of admitted necessity; the open ridicule with which were received the efforts of the Texas State Medical Association, through their committee, some of whom are gray-headed men, veterans in the cause of humanity. What striking evidence has the modern medical profession given to the world of its nobility? Have they founded any great charities, instituted any great reforms, done any practical benevolence? We remember with shame the utter failure, through niggardly stinginess and want of support, of the Physicians' Mutual Benefit Association, not only of Texas, but of several attempted in different parts of the Union. An attempt was made to secure one dollar donations from members of "our noble profession" for the widows and orphans of honorable men who had devoted their lives to the relief of others, even to the neglect of provision for their own loved ones. Instances of great distress come to our knowledge in Texas. An active, able and "noble" physician was cut down in the midst of a career of usefulness—to others—and his wife, tenderly raised and educated, had to hire out to get bread for herself and children. (We had the satisfaction of sending her $100, donated by one hundred really "noble" physicians.)

In light of the above, it is a source of gratification to observe that, judging from the following, there is an awakening to a realization of our remissness in this matter of practicing what we preach; a pricking of the conscience, which, we hope, will not "die in the bornin."

An effort is to be made by the great American Medical Association, to establish a practical benevolence—to make provision for the families of deceased members of our "noble prefession." At the last meeting, held in Washington, Dr. W. F. Horner, U. S. A., Va., offered the following, which was adopted:

WHEREAS, The Code of Ethics of this Association declares, Art. II., "The benefits accruing to the public directly and indirectly from the active and unweried beneficence of the profession," but—since its original organization has never considered any provision for the widows and orphans of deceased fellows.

WHEREAS, The success in the formation of Medical Aid Associations in our large cities and in one or more of the States of our Union has proved a practical scheme to succor the families of many a brother physician, stricken down in apparent success, who otherwise would be unprovided for; therefore, be it

*Resolved*, That a committee be appointed by the President of this Association to consider and report upon the expediency and practical advantages of a "Section of Benevolence" in connection with the work and aims of the Association, which seeks to ameliorate not only human suffering and to cure disease, but to enlarge the influence of the profession by showing regard for the needs of the widows and orphans of our deceased brethren.

And in this connection we "point with pride" also to the article elsewhere published in this issue (taken from the proceedings American Medical Association as published in the Journal of May 16) entitled, "Report of the Committee on the question of a Cabinet appointment of a Secretary of Public health." It is a "move in the right direction," and if its object can be consummated, it will be the first practical recognition of the medical profession as an element in the politics of America, or even as an integer of its population; its boasted "nobility" will then be put to the test, and an opportunity will be afforded it to establish its claims. That the opportunity will be improved, and the bureau made a practical machine for doing good, securing and enforcing wise and salutary laws, will depend on whether or not it becomes sucked into the maelstrom of politics, and is manipulated for revenue only; or to what extent it will be controlled by "bosses" and medical politicians. As Mr. Pecksniff would say: "An elligible opportunity now presents" to fix the seal to the claim that we are a "noble profession." The fate of the National Board of Health is yet green in the memory, and certain features of the M. H. S. rise up, unpleasant spectres, and warn us to beware of the politicians.

